# The membrane-distal regions of integrin α cytoplasmic domains contribute differently to integrin inside-out activation

**DOI:** 10.1038/s41598-018-23444-w

**Published:** 2018-03-22

**Authors:** Aye Myat Myat Thinn, Zhengli Wang, Jieqing Zhu

**Affiliations:** 1 Blood Research Institute, BloodCenter of Wisconsin, Milwaukee, WI 53226 USA; 2 Department of Biochemistry, Medical College of Wisconsin, Milwaukee, WI 53226 USA

## Abstract

Functioning as signal receivers and transmitters, the integrin α/β cytoplasmic tails (CT) are pivotal in integrin activation and signaling. 18 α integrin subunits share a conserved membrane-proximal region but have a highly diverse membrane-distal (MD) region at their CTs. Recent studies demonstrated that the presence of α CTMD region is essential for talin-induced integrin inside-out activation. However, it remains unknown whether the non-conserved α CTMD regions differently regulate the inside-out activation of integrin. Using α_IIb_β_3_, α_L_β_2_, and α_5_β_1_ as model integrins and by replacing their α CTMD regions with those of α subunits that pair with β_3_, β_2_, and β_1_ subunits, we analyzed the function of CTMD regions of 17 α subunits in talin-mediated integrin activation. We found that the α CTMD regions play two roles on integrin, which are activation-supportive and activation-regulatory. The regulatory but not the supportive function depends on the sequence identity of α CTMD region. A membrane-proximal tyrosine residue present in the CTMD regions of a subset of α integrins was identified to negatively regulate integrin inside-out activation. Our study provides a useful resource for investigating the function of α integrin CTMD regions.

## Introduction

Integrins are cell adhesion receptors composed of α and β subunits, each containing a large extracellular domain, a single transmembrane (TM) domain and usually a short cytoplasmic tail (CT). In human, the combinations of 18 α and 8 β subunits form 24 integrin heterodimers that play essential roles in numerous biological activities such as hemostasis, immune responses, and development^1^. Aberrant activation of integrin is associated with many pathological conditions including thrombosis, inflammatory diseases, and tumor-driven cell growth, metastasis, and angiogenesis^2–4^. Therefore, tight regulation of integrin activation is important for normal integrin function. A unique feature of integrins is that they can transmit signals bidirectionally across the cell membrane, so called inside-out and outside-in signaling^5,6^. In the inside-out direction, the activating signals impinge on the integrin CT to transform integrin from a resting to an active state by inducing large-scale conformational changes of the extracellular domain^7^. In the outside-in direction, ligand binding to the extracellular domain of active integrin also induces long-range conformational changes that are transmitted to the CT to provoke the association and activation of the kinases and adaptor molecules in the cytosol^8,9^. As such, acting as both the receiver and the transmitter of signals, integrin CT is pivotal in integrin activation and signaling.

**Figure 1:**
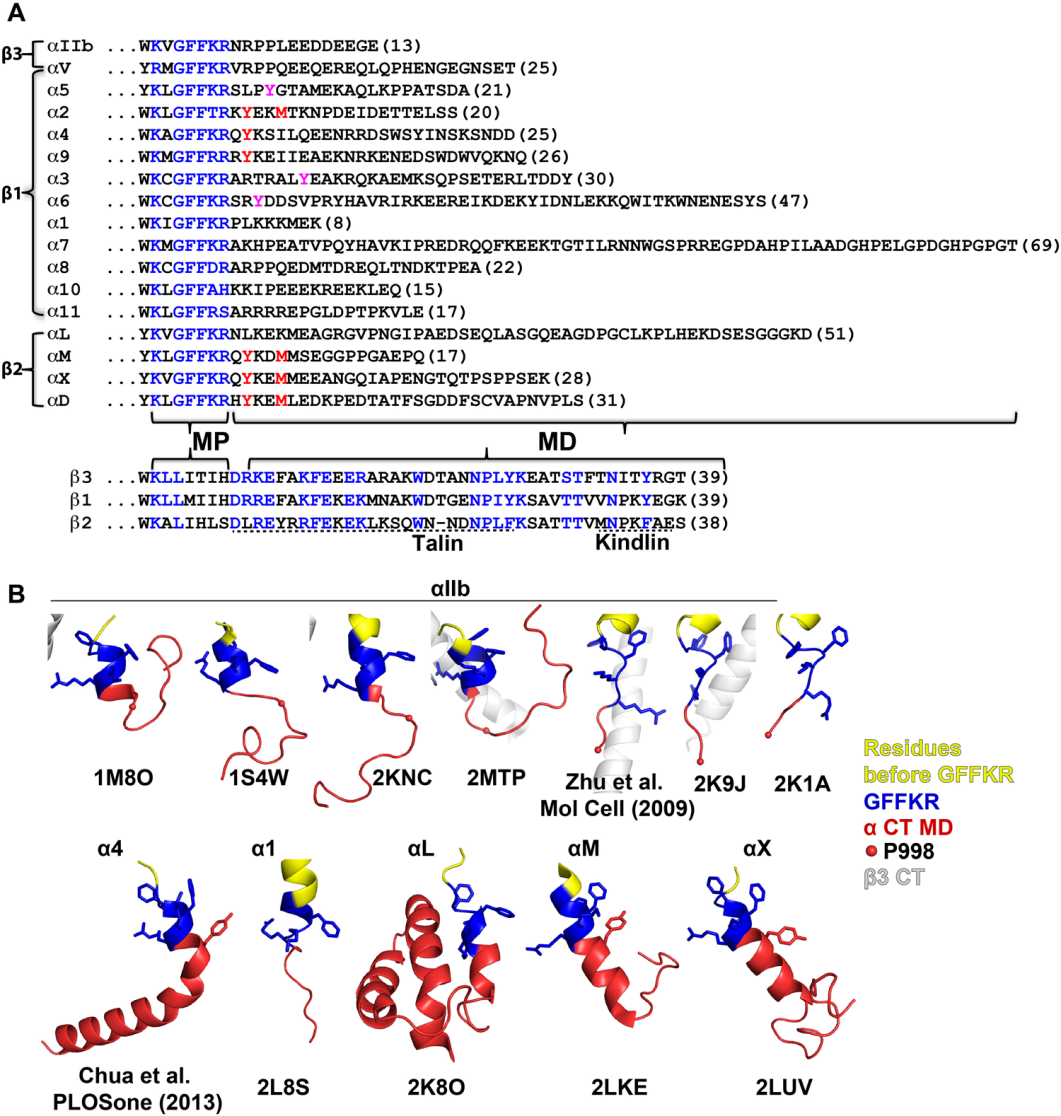
Sequence and structure diversity at the membrane -distal region of α integrin cytoplasmic domain. **(A)** Sequence alignment of α and selected β cytoplasmic tails (CTs) of human integrins. The membrane-proximal (MP) and the membrane-distal (MD) regions are indicated. Highly conserved residues are in blue. Tyrosine and methionine residues in the MD region that are conserved within a subset of α integrins are shown in red. Other tyrosine residues of interest that are adjacent to the GFFKR motif in several α subunits are shown in magenta. The lengths of the MD regions are in parentheses. The binding sites of talin and kindlin on integrin β CTs are indicated with dashed lines. **(B)** Comparison of the reported α integrin CT structures. PDB codes or references are shown below the corresponding structures. The structures are color-coded as indicated on the right. All the structures are superimposed onto the α_IIb_ structure (PDB code 1M8O) based on the GFFKR region and presented separately. Side chains of the GFFKR motif and the conserved tyrosine residues in the MD regions are shown as sticks.

Largely based on the studies of β_3_, β_2_, and β_1_ integrins, great progress has been made in understanding how the β integrin CT contributes to integrin activation^6,10^. Most of the β integrin CTs contain the conserved binding motifs for the common integrin activators, talin and kindlin (Fig. 1A). Structural and functional studies suggested that binding of talin and kindlin to β integrin CT induces integrin activation by disrupting the α-β interactions at the TM and the CT domains^10^, which in turn leads to conformational changes of the extracellular domain^5^. A functional role of the α integrin CT in integrin activation had been focused on the highly conserved Gly-Phe-Phe-Lys-Arg (GFFKR) motif at the membrane proximal (MP) region (Fig. 1A), which helps maintain the α-β CT associations^11,12^. Notably, the membrane-distal (MD) regions of the α integrin CT differ significantly in both amino acid sequence and length (Fig. 1A) and their roles in integrin activation and signaling remain ill defined. Accordingly, the current available structures of α integrin CTs also show conformational diversity at the MD regions (Fig. 1B). Moreover, even the same α_IIb_ integrin CTMD region shows different conformations among the reported structures (Fig. 1B). Recent studies from our and other groups demonstrated that the presence of α integrin CTMD region is essential in talin/kindlin induced integrin inside-out activation^13,14^. In addition, our study also showed that the length and amino acids of the α integrin CTMD region might be important in regulating integrin inside-out activation^13,14^. Given that one β integrin subunit such as β_3_, β_2_, or β_1_ usually heterodimerizes with more than one α subunits (Fig. 1A), an intriguing question is whether the diverse α CTMD regions contribute differently to integrin activation and signaling, which may determine the specific and diverse integrin functions.

In this study, using the platelet-specific α_IIb_β_3_, leukocyte-specific α_L_β_2_, and the ubiquitously expressed α_5_β_1_ as model integrins, we attempted to compare the effect of 17 out of 18 total α integrin CTMD regions on integrin inside-out activation. Our study revealed that the α CTMD regions contribute differently to talin head (TH)-induced integrin activation, evidenced by different levels of ligand binding and conformational changes. This was at least in part determined by the presence of specific residues in the α CTMD regions. Potential mechanisms by which the α CTMD regions participate in integrin activation were discussed.

## Results

### Design and generation of chimeric α integrins to examine the contributions of the diverse α CTMD regions in integrin inside-out activation

**Figure 2:**
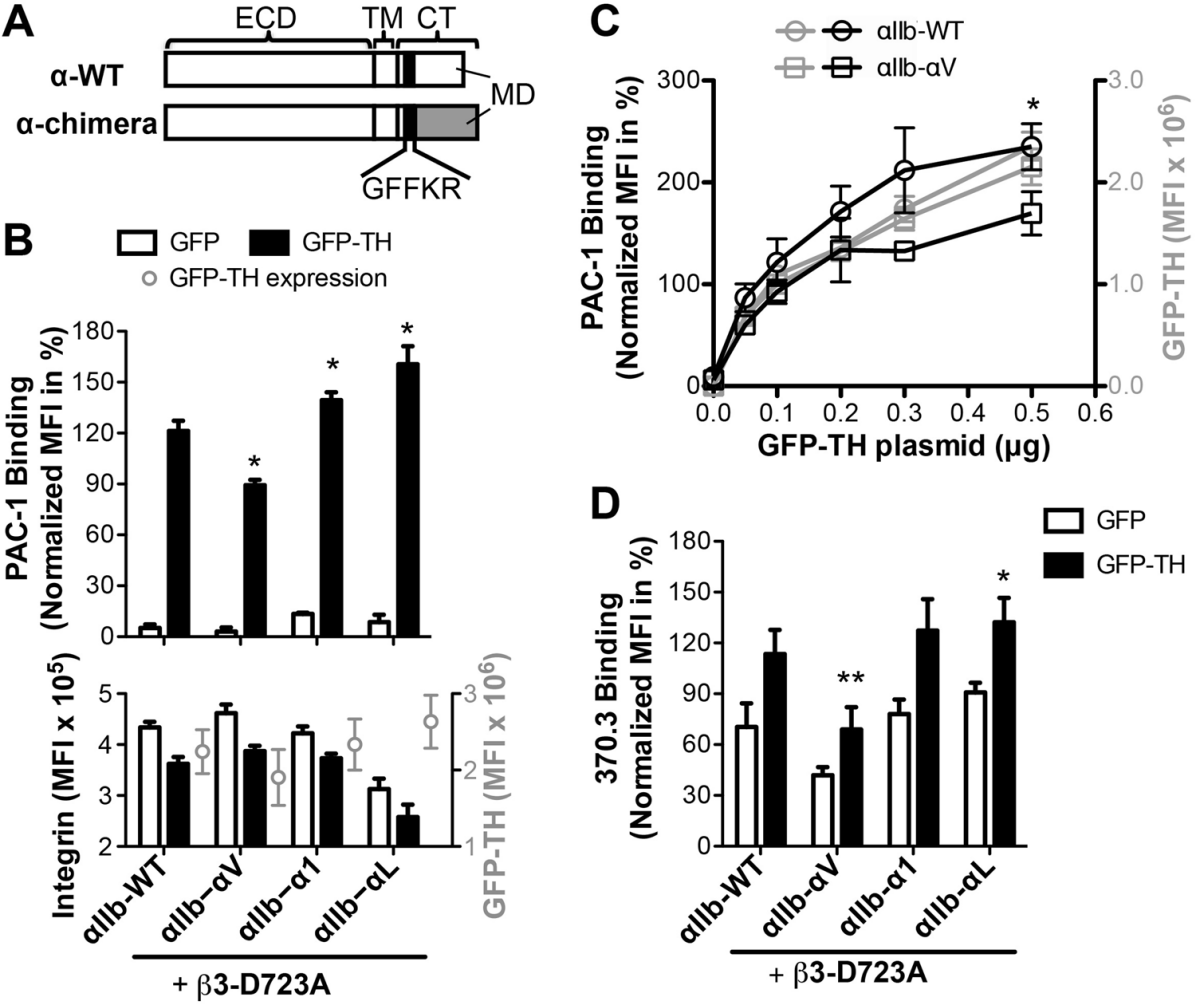
Integrin α_IIb_-chimeras bearing various α CTMD regions responded differently to talin head (TH)-induced integrin activation. (**A**) Design of α integrin chimera. Integrin α chimera is constructed by replacing the MD region of a model α integrin with those of indicated α integrins. (**B**) TH-induced binding of the ligand-mimetic mAb PAC-1 to the α_IIb_ chimeras. (**C**) PAC-1 binding to α_IIb_-WT and α_IIb_-α_V_ in response to the different expression levels of GFP-TH. PAC-1 binding was measured with the HEK293FT cells transfected with the α_IIb_ integrins plus the β_3_-D723A mutant and the indicated amounts of GFP-TH plasmids. (**D**) TH-induced binding of the active conformation-specific mAb 370.3 to the α_IIb_ chimeras. Binding of the mAbs was measured by flow cytometry with HEK293FT cells co-transfected with the indicated integrin constructs and GFP or GFP-TH. The GFP and integrin double-positive cells were analyzed. β_3_-D723A was used to increase the sensitivity of the assay. Data are presented as the MFI of the mAb binding normalized to the MFI of integrin expression. Data are presented as mean ± s.e.m. (n ≥ 3) and two-tailed Student’s t-test was performed to compare the α_IIb_-chimeras to α_IIb_-WT under the GFP-TH condition in B and D or under same GFP-TH concentration in C; *P < 0.05, **P < 0.01. Integrin and GFP-TH expression levels were presented in MFI in the lower panel in B. n = 2 for α_IIb_-α_1_ in D.

Studies from our group and others have demonstrated the requirement of the presence of α CTMD region in integrin inside-out activation^13^. However, it remains elusive whether and/or how the diverse CTMD regions regulate integrin activation. It has been shown that the α CTMD regions also contribute to maintaining integrin in the resting state possibly by interacting with the β CT^13–15^. Therefore, a simple mutagenesis or replacement of the α CTMD region with an irrelevant sequence may result in complicated and uninterpretable results. To address this question, we took the advantage that one β subunit usually pairs with more than one α subunits. For example, β_3_, β_2_, and β_1_ subunit can heterodimerize with 2, 4, and 12 different α subunits, respectively (Fig. 1A). Furthermore, *in vitro* activation assays of α_IIb_β_3_, α_L_β_2_, and α_5_β_1_ have been very well established^13,16,17^. Therefore, we used α_IIb_, α_L_, and α_5_ as model α integrins, in which their CTMD regions were replaced by those of α subunits that can pair with β_3_, β_2_, and β_1_ subunits, respectively (Fig. 2A). In such way, we generated the α chimeras that are denoted as α_IIb_-α_V,_ α_L_-α_X_, α_L_-α_D_, α_L_-α_M_, α_5_-α_V_, α_5_-α_1_, α_5_-α_2_, α_5_-α_3_, α_5_-α_4_, α_5_-α_6_, α_5_-α_7_, α_5_-α_8_, α_5_-α_9_, α_5_-α_10_ and α_5_-α_11._ When these α_IIb_, α_L_, and α_5_ chimeras are co-expressed with β_3_, β_2_, and β_1_ subunits, respectively, the native associations of α and β CT domains are maintained. In addition, comparisons can be made among the α chimeras that share the same β subunit. Any differences seen in the integrin inside-out activation assay would attribute to the diverse CTMD regions. These 15 chimeras together with the wild type (WT) α_IIb_, α_L_, and α_5_ subunits allow our study to cover the CTMD regions of 17 out of 18 human α integrins.

### Integrin α_IIb_-chimeras bearing various α CTMD regions respond differently to talin head (TH) stimulation

The β_3_ integrin’s partners α_IIb_ and α_V_ subunits share 6 consensus residues at their CTMD regions, but the α_V_ CTMD region is about two times longer than that of the α_IIb_ (Fig. 1A). We have shown that complete deletion of the CTMD regions of α_IIb_ and α_V_ subunits abolished TH-induced α_IIb_β_3_ and α_V_β_3_ activation^13^. Here, we asked whether the CTMD regions of α_IIb_ and α_V_ subunits could be exchangeable and whether they could exert different effect on β_3_ integrin inside-out activation. The ligand-mimetic mAb PAC-1 was used to access the α_IIb_β_3_ activation induced by the overexpression of GFP-TH in the presence of the β_3_ cytoplasmic mutation β_3_-D723A, which has been shown to greatly enhance the responsiveness of TH-induced α_IIb_β_3_ activation^13^. When the CTMD region of α_IIb_ was replaced by the α_V_ CTMD region, the α_IIb_-α_V_/β_3_-D723A chimeric integrin still remained responsive to GFP-TH-induced activation (Fig. 2B). However, the activation of α_IIb_-α_V_ was significantly decreased compared with the α_IIb_-WT (Fig. 2B). The reduced activation of α_IIb_-α_V_ chimera was not due to the differences in GFP-TH expression since the lower activity of α_IIb_-α_V_ compared with α_IIb_-WT was consistently seen at various levels of GFP-TH expression (Fig. 2C). As a comparison, we replaced the α_IIb_ CTMD region with those of α_1_ and α_L_ integrins that do not heterodimerize with β_3_ integrin. Remarkably, the presence of both the α_1_ and α_L_ CTMD regions significantly enhanced the GFP-TH-induced activation of α_IIb_β_3_ integrin (Fig. 2B). The increased activation of α_IIb_-α_1_ and α_IIb_-α_L_ was also obvious in the absence of TH expression (Fig. 2B), indicating that the mismatch of the α_IIb_ CTMD mutant with the β_3_ CT renders α_IIb_β_3_ more active than the wild type. This may be due to the destabilization of α_IIb_-β_3_ CT interaction, being consistent with the previous observations that the α CTMD regions contribute to maintaining integrin in the resting state^13,14^. The expression level of α_IIb_-α_L_ was decreased possibly due to the high integrin activity (Fig. 2B), which is commonly seen among the active integrin mutants^13^. We next asked whether the replacement of α_IIb_ CTMD region affects the TH-induced conformational change of α_IIb_β_3_ integrin. The active conformation-specific mAb 370.3 was used to report the extension of α_IIb_ integrin. Consistent with the PAC-1 binding assay, the α_IIb_-α_V_ chimera showed decreased while the α_IIb_-α_1_ and α_IIb_-α_L_ chimeras showed increased binding of mAb 370.3 either in the presence or absence of TH expression (Fig. 2D). This data demonstrates that the CTMD regions of α_IIb_ and α_V_ are not completely interchangeable. They can exert different effect on β_3_ integrin activation at least in part through regulating the conformational change of integrin.

### Replacing the α_L_ CTMD region with that of α_X_, α_D_ or α_M_ subunit reduced TH-mediated α_L_β_2_ integrin activation

**Figure 3:**
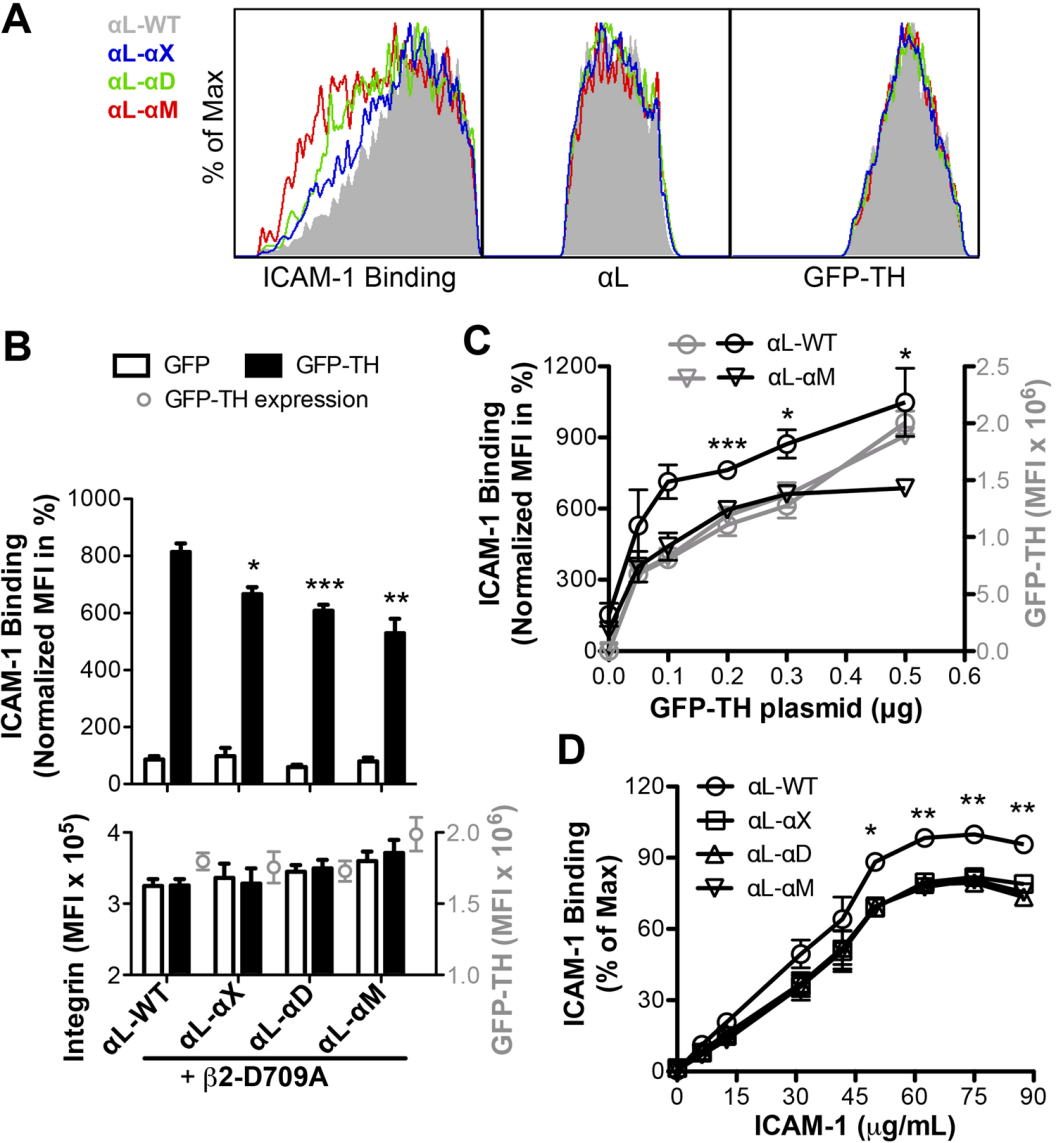
Integrin α_L_-chimeras bearing the α_X_, α_D_ or α_M_ CTMD regions show lower levels of TH-induced integrin activation than α_L_-WT. (**A)** Representative overlaid flow cytometry plots of ICAM-1 binding, α_L_ and GFP-TH expression in the log scale. HEK293FT cells were co-transfected with the indicated α_L_-chimeras and β_2_-D709A mutant plus GFP (plots not shown) or GFP-TH. The integrin and GFP-TH double-positive cells were gated for plotting the ICAM-1 binding and the expression of integrin and GFP-TH. **(B)** TH-induced ICAM-1 binding (quantitative data of A). Integrin and GFP-TH expression were presented in MFI in the lower panel. **(C)** ICAM-1 binding of α_L_-WT and α_L_-α_M_ chimera in response to the different levels of GFP-TH expression. α_L_ integrins were co-transfected with β_2_-D709A and the indicated amounts of GFP-TH plasmids into HEK293FT cells. For B and C, data are presented as the ICAM-1 MFI normalized to α_L_ MFI and shown as mean ± s.e.m. (n ≥ 3). Two-tailed Student’s t-test was performed to compare the α_L_-chimeras with α_L_-WT in the presence of GFP-TH in B or under same GFP-TH concentration in C. *P < 0.05; **P < 0.01; ***P < 0.001. **(D)** Dose response curves of ICAM-1 binding to α_L_-WT and α_L_-chimeras. HEK293FT cells were transfected with the α_L_ integrins plus β_2_-D709A and GFP-TH. Different concentrations of ICAM-1 were used for the binding assay. Data are presented as the percentage of maximum ICAM-1 binding of each experimental repeat and shown as mean ± s.e.m. (n ≥ 3). Two-tailed Student’s t-test was performed to compare the α_L_-chimeras with α_L_-WT under the same ICAM-1 concentration; *P < 0.05; **P < 0.01. ICAM-1 binding was statistically lower for all α_L_-chimeras compared to α_L_-WT at the indicated ICAM-1 concentrations, but the analyses were shown for comparison between the α_L_-WT and α_L_-α_X_ chimera.

Integrin β_2_ subunit forms heterodimers with α_L_, α_M_, α_X_, and α_D_ subunits. α_L_ has the longest while α_M_ has the shortest CTMD sequence among the four subunits (Fig. 1A). The NMR structures of α_L_, α_M_ and α_X_ CTs show great structural heterogeneities at their MD regions (Fig. 1B). In addition, we found that deletion of the α_L_ CTMD region abolished, while truncation of the α_L_ CTMD region dampened TH-induced α_L_β_2_ activation^13^, arguing a potential regulatory role of the CTMD region. Similar to the α_IIb_ chimeras, we made the α_L_ chimeras by replacing the α_L_ CTMD region with that of α_M_, α_X_ or α_D_ subunit. Surprisingly, in the TH-induced ICAM-1 binding assay, all the α_L_-α_M_, α_L_-α_X,_ and α_L_-α_D_ chimeras showed significantly reduced ICAM-1 binding compared with the WT α_L_ when co-expressed with the β_2_-D709A mutation (Fig. 3A,B). The β_2_-D709A mutation was used to increase the sensitivity of our assay by greatly enhancing TH-induced α_L_β_2_ activation as shown in our previous study^13^. The expression levels of integrin and GFP-TH were comparable among the α_L_ transfections (Fig. 3B). Furthermore, as shown for the α_L_-α_M_ chimera in the GFP-TH titration assay, the reduced ICAM-1 binding was obvious when the GFP-TH expression reached a certain level and became independent of the expression level of GFP-TH (Fig. 3C). Similar results were obtained with the α_L_-α_D_ chimera (data not shown). In addition, although all the α_L_ constructs exhibited increased TH-induced ICAM-1 binding with the increase of ICAM-1 concentration, all the α_L_ chimeras consistently showed reduced ICAM-1 binding at all the ICAM-1 concentrations tested (Fig. 3D). These data demonstrated that the reduced activation of α_L_ chimeras was due to the replacement of CTMD region and might attribute to a common feature of α_M_, α_X_, and α_D_ CTMD regions.

### A conserved tyrosine residue in the CTMD regions of α_M_, α_X_, and α_D_ subunits negatively regulates the inside-out activation of β_2_ integrin

**Figure 4:**
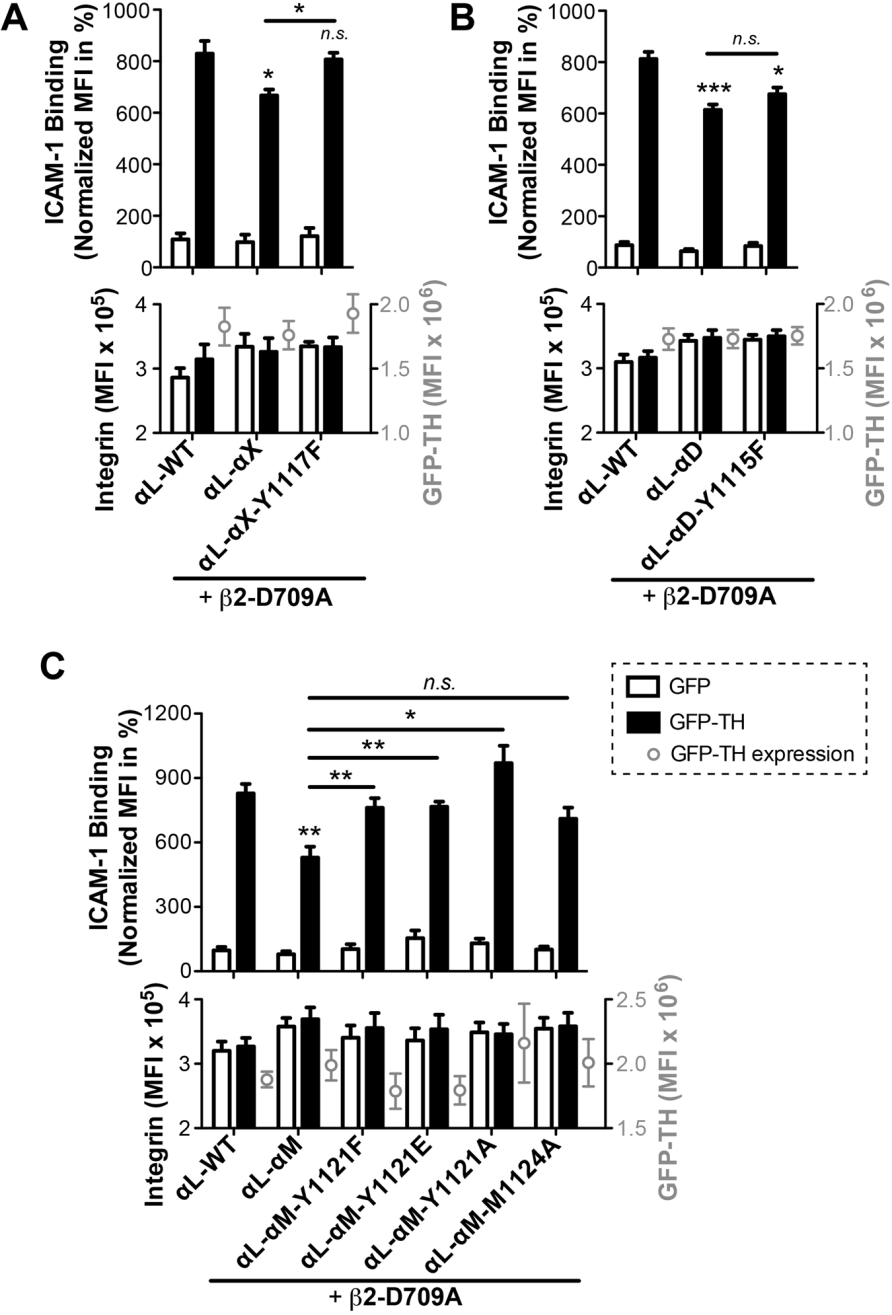
A conserved tyrosine residue within the MD regions of α_X_, α_M_, and α_D_ negatively regulates TH-induced integrin activation. (**A–C)** TH-induced ICAM-1 binding. The relatively conserved tyrosine was mutated to phenylalanine in all α_L_-chimeras (**A**–**C**), and to glutamic acid or alanine in the α_L_-α_M_ chimera (**C**). A conserved methionine was also mutated to alanine in the α_L_-α_M_ chimera (**C**). Binding of ICAM-1 was measured by flow cytometry with HEK293FT cells co-transfected with the indicated α_L_ constructs plus β_2_-D709A and GFP or GFP-TH. The GFP and integrin double-positive cells were analyzed. Data are presented as the MFI of the ICAM-1 binding normalized to integrin expression and shown as mean ± s.e.m. (n ≥ 3). Two-tailed Student’s t-test was performed to compare the α_L_-chimeras or their mutants to α_L_-WT under the GFP-TH condition, or as indicated (*P < 0.05; **P < 0.01; ***P < 0.001; *n*.*s*., not significant). Integrin and GFP-TH expression levels were presented in the lower panel.

Sequence alignment revealed a conserved tyrosine residue at the second position of all the CTMD regions of α_M_, α_X_, and α_D_ subunits (Fig. 1A). We asked whether this tyrosine contributes to the reduced activation of α_L_ chimeras. We first tested the tyrosine mutation on α_L_-α_X_ chimera with the co-expression of β_2_-D709A mutant. Compared with the α_L_-α_X_, the tyrosine to phenylalanine mutation at the α_X_ CTMD region, α_L_-α_X_-Y1117F, significantly increased the TH-induced ICAM-1 binding and restored it to the WT α_L_ level (Fig. 4A). Similarly, the α_L_-α_D_-Y1115F mutation also increased the TH-induced ICAM-1 binding although it was not to the WT level and not statistically significant (Fig. 4B). We further tested the tyrosine mutation on the α_L_-α_M_ chimera. Mutating the tyrosine in the α_M_ CTMD sequence to phenylalanine (Y1121F), glutamic acid (Y1121E), and alanine (Y1121A) all significantly increased the TH-induced ICAM-1 binding of α_L_-α_M_ chimera (Fig. 4C). Interestingly, the Y1121A mutation exerted a higher level of activation than the Y1121F and Y1121E mutations (Fig. 4C), indicating that both the bulky side chain and the hydroxyl group of tyrosine are important for its negative effect on integrin activation. Sequence alignment also shows a conserved methionine at the fifth position of all the α_M_, α_X_, and α_D_ regions (Fig. 1A). We tested whether this methionine residue contributes to the negative effect of CTMD region using the α_L_-α_M_ chimera. We found that the α_L_-α_M_-M1124A mutation did not significantly increase TH-induced ICAM-1 binding (Fig. 4C). The expression levels of integrin and GFP-TH were comparable among the transfections within the same experimental group (Fig. 4A–C). These data clearly demonstrate that the negative effects of the CTMD regions of α_M_, α_X_, and α_D_ subunits on TH-induced β_2_ integrin activation at least in part attribute to the presence of a conserved tyrosine.

### The position of the tyrosine mutation at the CTMD region is critical for its negative regulation of the α_L_β_2_ integrin inside-out activation

**Figure 5:**
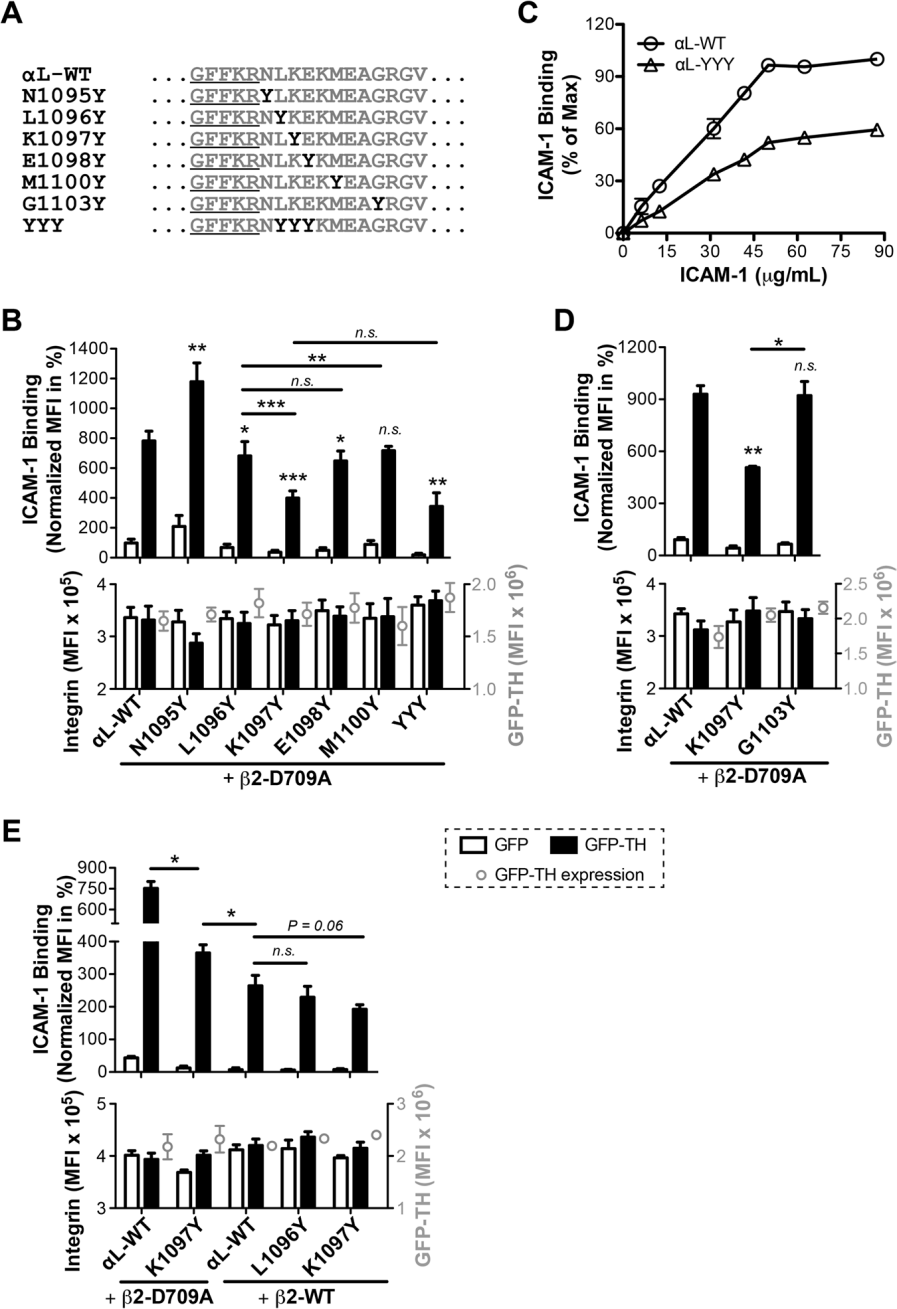
The position of a tyrosine mutation at the α_L_ CTMD region determines its negative effect on α_L_ integrin inside-out activation. (**A**) Tyrosine mutations introduced into the α_L_ CTMD region. (**B**,**D**-**E**) TH-induced ICAM-1 binding of the α_L_ tyrosine mutations co-expressed with the β_2_-D709A mutant (**B**,**D**) or β_2_-WT (**E**). Binding of ICAM-1 was measured by flow cytometry with HEK293FT cells co-transfected with the indicated integrin constructs and GFP or GFP-TH. The GFP and integrin double-positive cells were analyzed. ICAM-1 binding is presented as the MFI of ICAM-1 normalized to integrin expression, and shown as mean ± s.e.m. (n ≥ 3). Two-tailed Student’s t-test was performed to compare the α_L_ mutants to α_L_-WT under GFP-TH condition, or as indicated. *P < 0.05; **P < 0.01; ***P < 0.001; n.s., not significant. Integrin and GFP-TH expression levels were presented in the lower panel. (**C**) Dose response curves of ICAM-1 binding to selected tyrosine mutant of α_L_ integrin. The α_L_ constructs were co-expressed with β_2_-D709A and GFP-TH in HEK293FT cells. ICAM-1 binding is measured by flow cytometry and analyzed for the GFP and integrin double-positive cells. Data are presented as the percentage of maximum ICAM-1 binding of each experimental repeat and shown as mean ± s.e.m. (n = 2).

Interestingly, there is only one tyrosine in the CTMD region of α_M_, α_X_, or α_D_ subunit and no tyrosine in the α_L_ CTMD region (Fig. 1A). To further demonstrate the important regulatory role of a tyrosine residue in the CTMD region, we performed a tyrosine scanning mutagenesis for the α_L_ CTMD region. A tyrosine mutation was placed at the 1^st^, 2^nd^, 3^rd^, 4^th^, or 6^th^ position of the CTMD region after the conserved GFFKR motif (Fig. 5A). As shown in the TH-induced ICAM-1 binding assay, the tyrosine mutation at the 1^st^ position, α_L_-N1095Y, significantly enhanced ICAM-1 binding (Fig. 5B). This is probably due to the disturbance of α-β association at the GFFKR motif, which was known to be important in maintaining integrin in the resting state^11^. In contrast, the tyrosine mutation at the 2^nd^ position, α_L_-L1096Y, which is equivalent to the native tyrosine of the α_X_, α_D_ and α_M_ CTMD regions, significantly reduced TH-induced ICAM-1 binding (Fig. 5B). This is consistent with the above data. Remarkably, the tyrosine mutations at the 3^rd^ and 4^th^ positions, α_L_-K1097Y and α_L_-E1098Y, also significantly reduced ICAM-1 binding (Fig. 5B). However, the tyrosine mutation at the 6^th^ position, α_L_-M1100Y, had no significant effect on ICAM-1 binding (Fig. 5B). Among the single tyrosine mutations, the tyrosine mutation at the 3^rd^ position, α_L_-K1097Y, had the most negative effect (Fig. 5B). We next asked whether the presence of multiple tyrosine mutations at the CTMD region has a synergistic effect on its negative regulation of integrin activation. A triple tyrosine repeat was introduced into the 2^nd^ to 4^th^ positions of α_L_ CTMD region (Fig. 5A). The α_L_-YYY mutation significantly reduced ICAM-1 binding compared with the wild type, but had no significant difference with the single α_L_-K1097Y mutation (Fig. 5B). Furthermore, the negative effect of the tyrosine mutation did not depend on the ICAM-1 concentration (Fig. 5C). These data demonstrate that the presence and the position but not the number of tyrosine mutations at the CTMD region are important in the negative regulation of α_L_β_2_ activation.

The α_L_-K1097Y mutation coincidently formed an Yxxɸ motif (x is any amino acid, ɸ is hydrophobic residue), which has been found recently in a subset of α integrins to play a role in the regulation of integrin endocytosis^18^. It has been shown that the presence but not the position of the Yxxɸ motif is important for its function in integrin endocytosis^18^. To test whether the negative effect of α_L_-K1097Y mutation was due to the formation of Yxxɸ motif, we generated another tyrosine mutation, α_L_-G1103Y, which formed an Yxxɸ motif (Fig. 5A). In contrast to the α_L_-K1097Y mutation, the α_L_-G1103Y mutation showed no difference with the α_L_-WT in ICAM-1 binding (Fig. 5D), suggesting that the negative effect of α_L_-K1097Y mutation is not due to the presence of an Yxxɸ motif.

In our TH-induced ICAM-1 binding assay, all the α_L_ constructs were co-expressed with the β_2_-D709A mutation. To demonstrate that the differences we observed among the α_L_ mutations in the integrin activation assay are not due to the presence of the β_2_-D709A mutation, we did the same assay in the presence of β_2_-WT for several representative tyrosine mutations. The results show that all the selected α_L_ tyrosine mutations reduced TH-induced ICAM-1 binding although not as significant as in the presence of the β_2_-D709A mutation (Fig. 5E).

### The α CTMD regions contribute to integrin activation by regulating the conformational change of integrin

**Figure 6:**
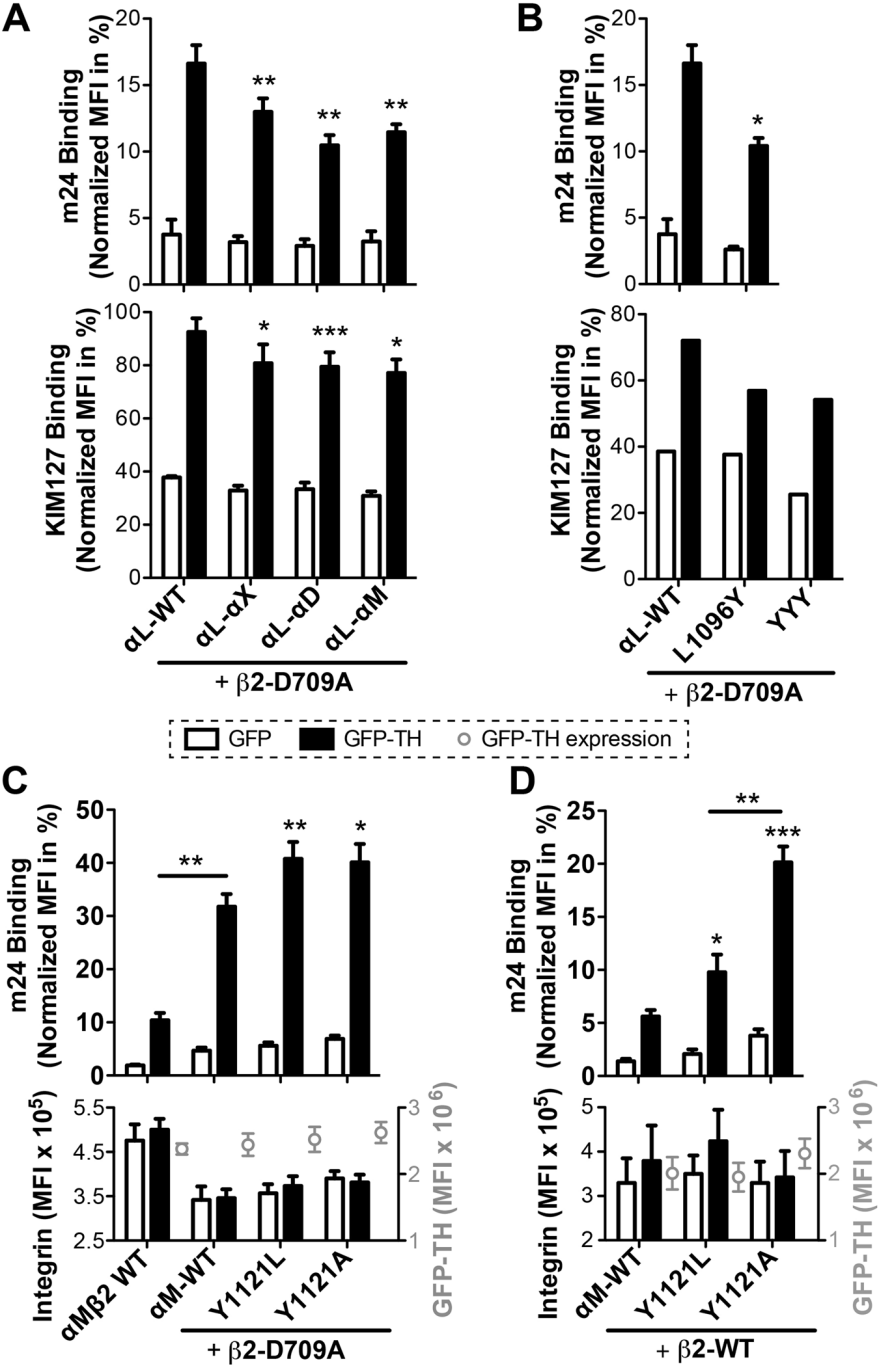
The α CTMD regions contribute to integrin activation by regulating the conformational change of integrin. (**A**,**B)** TH-induced integrin conformational change. Binding of mAb KIM127 (reports integrin extension) or m24 (reports integrin headpiece opening) was assessed with (**A**) α_L_-chimeras, or with (**B**) selected α_L_ tyrosine mutants co-expressed with β_2_-D709A in HEK293FT cells. **(C**,**D)** TH-induced integrin conformational change of α_M_ integrin constructs co-expressed with β_2_-D709A or β_2_-WT. Binding of m24 or KIM127 mAb was measured by flow cytometry with HEK293FT cells co-transfected with the indicated integrin constructs and GFP or GFP-TH. Data are presented as the MFI of bound m24 or KIM127 normalized to the MFI of integrin expression, and shown as mean ± s.e.m. (n ≥ 3). Two-tailed Student’s t-test was performed to compare the α_L_ mutants to α_L_-WT in A and B, or to compare the α_M_ mutants to α_M_-WT, or as indicated under the GFP-TH condition in C and D (*P < 0.05; **P < 0.01; ***P < 0.001). Integrin and GFP-TH expression levels were presented in MFI in lower panel of C and D. One representative experiment was shown for the KIM127 binding in B.

TH-induced integrin activation is coupled with the large-scale conformational changes of integrin extracellular domain^5,10^. We used two mAbs, KIM127 and m24, which report β_2_ integrin extension and headpiece opening, respectively^19^, to test whether the mutagenesis of α_L_ CTMD region affect TH-induced α_L_β_2_ conformational change. Consistent with the ICAM-1 binding assay, the α_L_-α_M_, α_L_-α_X,_ and α_L_-α_D_ chimeras all significantly reduced TH-induced binding of both m24 and KIM127 mAbs when compared with the α_L_-WT (Fig. 6A). Similarly, the α_L_ tyrosine mutations, α_L_-L1096Y and α_L_-YYY, also decreased the TH-induced m24 or KIM127 binding to α_L_β_2_ (Fig. 6B). These data demonstrate that the α CTMD region contribute to integrin inside-out activation through regulating the large-scale conformational changes.

Having found that introducing a tyrosine residue into the specific position of α_L_ CTMD region negatively regulates α_L_β_2_ ligand binding and conformational change, the next question is whether the native tyrosine residue present in the α_M_, α_X,_ or α_D_ CTMD region plays a role in regulating the activation of these integrins. To answer this question, we performed the TH-induced activation assay for α_M_β_2_ integrin by detecting the β_2_ integrin headpiece opening using m24. Consistent with the α_L_β_2_, the presence of β_2_-D709A mutation greatly enhanced TH-induced binding of m24 to α_M_β_2_ (Fig. 6C). When replacing the conserved tyrosine at the 2^nd^ position of α_M_ CTMD region (Y1121) with either leucine or alanine, the TH-induced binding of m24 was further increased significantly in the presence of β_2_-D709A (Fig. 6C). The same effect was found when the α_M_-Y1121L or α_M_-Y1121A was co-expressed with the β_2_-WT (Fig. 6D). Interestingly, the enhanced activation by α_M_-Y1121A is more obvious than α_M_-Y1121L when they were co-expressed with β_2_-WT (Fig. 6D), again suggesting that the bulky side chain of tyrosine is important for the negative effect on integrin activation. The expression level of integrin or GFP-TH is comparable among the transfections (Fig. 6C,D). These data, in addition to the α_L_ tyrosine mutations that exerted the opposite effect on α_L_β_2_ integrin activation, clearly demonstrate that a specific tyrosine residue present in a subset of α integrin CTMD regions negatively regulates β_2_ integrin inside-out activation.

### Mutagenesis of the α_IIb_ and α_L_ CTMD regions does not affect the TH binding to the integrin β_3_ and β_2_ CTs

**Figure 7:**
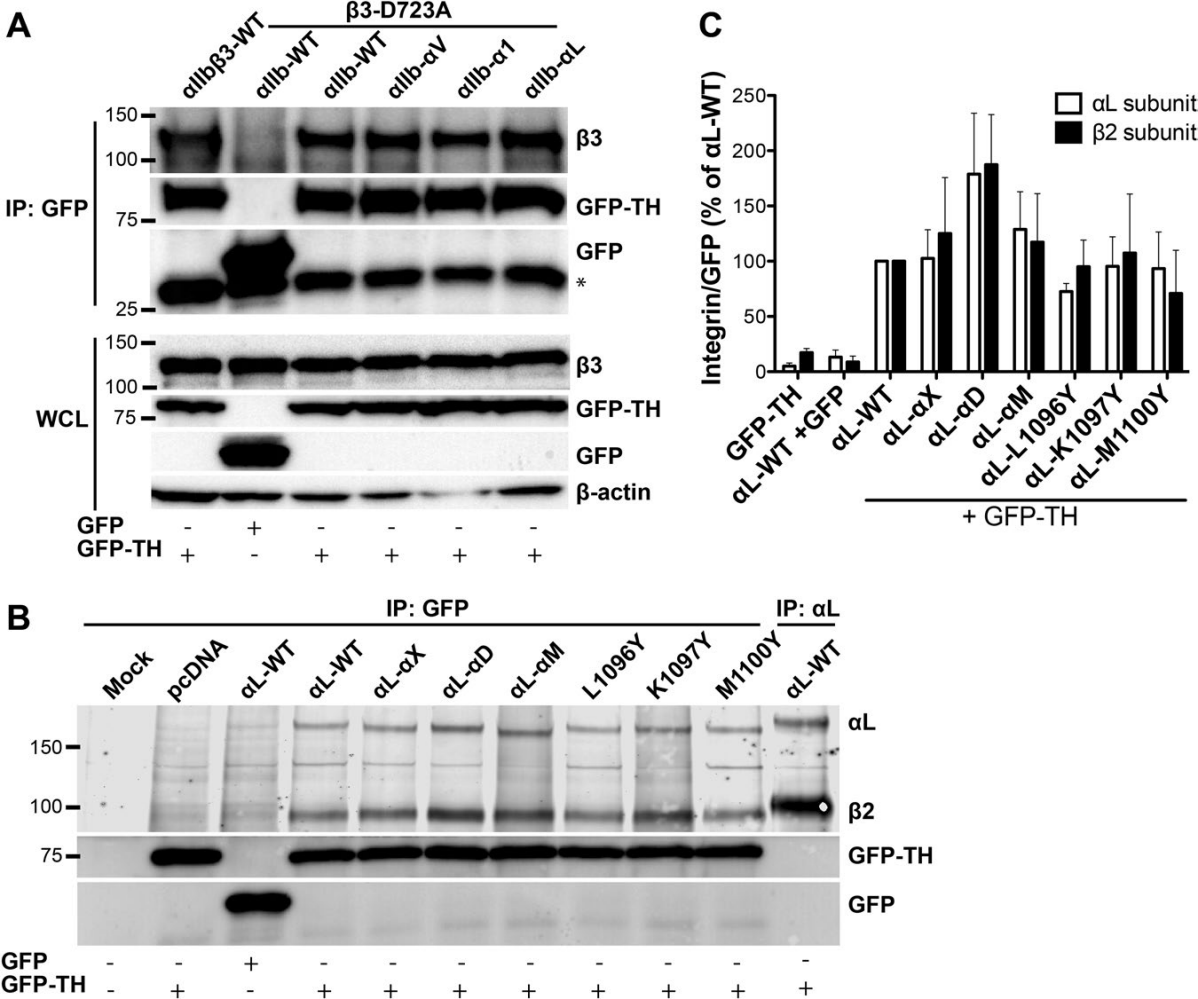
Swapping or mutating the residues in the CTMD region of integrin α_IIb_ or α_L_ does not dramatically affect the TH binding to the β integrin CT. (**A**) TH binding to β_3_ integrin CT in the presence of α_IIb_-chimeras. To detect the interaction between GFP-TH and integrin β_3_ CT, GFP-TH was immunoprecipitated using anti-GFP antibody from the lysates of HEK293FT cells transfected with GFP (control) or GFP-TH, plus indicated α_IIb_ and β_3_ integrin constructs. The associated integrin was detected by immunoblot with anti-β_3_ antibody. Expression level of β_3_, GFP or GFP-TH was accessed by immunoblots using whole cell lysates (WCL). β-actin was blotted as a loading control. The asterisk indicates a non-specific band. The membranes were cut and blotted separately. (**B**) TH binding to β_2_ CT in the presence of α_L_-chimeras or α_L_ tyrosine mutants. HEK293FT cells were co-transfected with the indicated α_L_ constructs plus β_2_-D709A and GFP or GFP-TH. The transfected cells were biotinylated prior to the anti-GFP immunoprecipitation as described in A or anti-α_L_ immunoprecipitation as a control. The GFP-TH associated integrin was detected by blotting with the IRDye® 800CW Streptavidin. The immunoprecipitated GFP or GFP-TH were detected by anti-GFP antibody. The results were from the same gel. The membrane was cut and blotted separately. (**C**) Quantitation of the blotting results of B. Integrin α_L_ or β_2_ signals were first normalized to the corresponding GFP or GFP-TH signals, and then presented as a percentage of the normalized α_L_-WT or β_2_ signal to the α_L_-WT control under the GFP-TH condition. Data are mean ± s.e.m. (n ≥ 3) except for the β_2_ signal with α_L_-M1100Y (n = 2). Please also see Fig. S1 for panels A and B.

Structural and functional studies have demonstrated that TH binds to the integrin β CT to induce integrin activation^10^. To test whether the α CTMD region contributes to integrin activation by affecting the TH association with the β CT, we did co-immunoprecipitation assay for the GFP-TH and integrin β subunit co-expressed with the α CTMD mutants. For the α_IIb_β_3_ transfectants, the cell lysates were precipitated with anti-GFP antibody and the associated β_3_ integrins were detected by the anti-β_3_ antibody. The β_3_ WT or β_3_-D723A was robustly detected in the anti-GFP pull-down only when the GFP-TH but not GFP was present, demonstrating the specific interaction between TH and β_3_ subunit (Figs 7A and S1). The expression levels of GFP-TH and β_3_ integrin were comparable among the transfectants according to the Western blot of whole cell lysate (Figs 7A and S1). No obvious differences for the TH-bound β_3_-D723A were observed among the α_IIb_ WT and α_IIb_ chimeras, suggesting that swapping the α_IIb_ CTMD region with that of α_V_, α_1_, or α_L_ does not affect the association of TH with β_3_ subunit.

We next performed the same assay for the α_L_ CTMD mutations. To simultaneously detect both the α_L_ and β_2_ subunits in the anti-GFP pull-down assay, we did the cell surface biotinylation before lysing the cells for co-immunoprecipitation. The presence of α_L_ and β_2_ bands was confirmed by anti-α_L_ pull-down using the anti-α_L_ specific mAb TS2/4. As shown in Fig. 7B, two bands that correspond to the α_L_ and β_2_ subunits were readily detected in the anti-GFP pull-down only when both the α_L_β_2_ and GFP-TH were co-expressed. No α_L_ and β_2_ bands were detected in the anti-GFP pull-down in the transfections of GFP-TH alone or α_L_β_2_ plus GFP (Figs 7B and S1). The expression levels of integrin and GFP-TH were comparable among the transfections detected by flow cytometry (data not shown). To compare the association of GFP-TH and the α_L_β_2_ constructs, we quantified the Western blots by normalizing the α_L_ and β_2_ signals to the GFP-TH signals (Fig. 7C). Compared with the α_L_ WT, only the α_L_-α_D_ chimera shows an obvious increase in the association of integrin and GFP-TH (Fig. 7C). This is in contrast to the decrease in TH-induced α_L_β_2_ activation as shown above. No significant differences were found among the α_L_ WT, α_L_-α_X_, α_L_-α_M_, and the α_L_ tyrosine mutations, α_L_-L1096Y, α_L_-K1097Y, and α_L_-M1100Y (Fig. 7C). These data indicate that the regulatory function of α CTMD region on integrin activation should not be due to the effect on the TH and β CT association.

### Integrin α_5_-chimeras bearing various α CTMD regions respond differently to TH stimulation when paired with the same integrin β_1_ subunit

**Figure 8:**
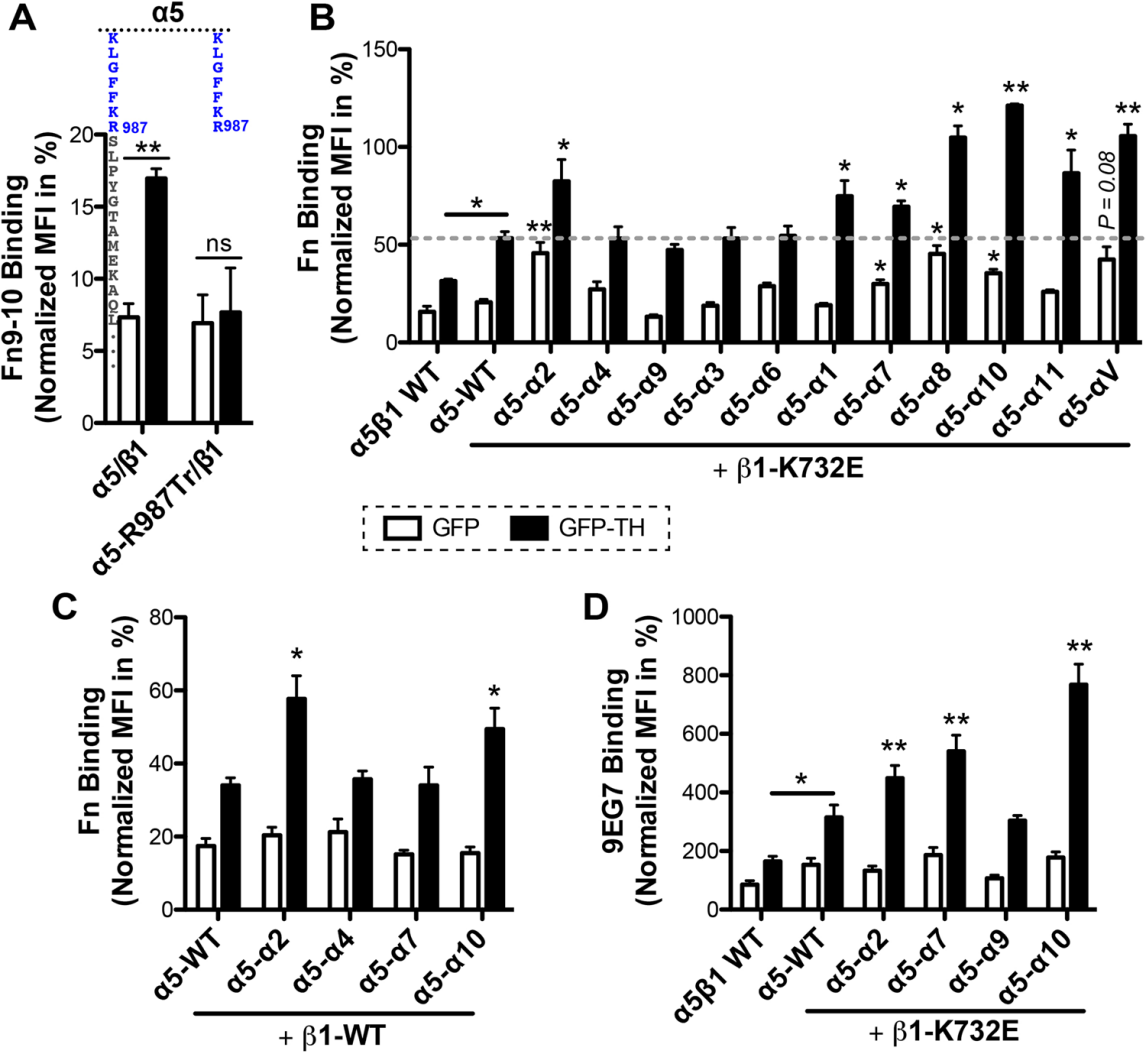
Comparison of the contribution of different α CTMD regions on TH-induced integrin activation using α_5_β_1_ integrin as a platform. (**A**) Deletion of the α_5_ CTMD region abolished TH-induced α_5_β_1_ activation. Binding of the fibronectin type III domains 9–10 fragment (Fn9–10) was measured by flow cytometry with CHO-B2 cells transfected with the indicated α_5_β_1_ constructs plus GFP or GFP-TH. (**B**) Fibronectin (Fn) binding of the α_5_ chimeras co-expressed with β_1_-K732E. To make the α_5_ chimeras, the α_5_ CTMD region was replaced with those of α integrins that can pair with β_1_ subunit. (**C**) Fn binding of selected α_5_ chimeras co-expressed with β_1_-WT. (**D**) mAb 9EG7 binding of selected α_5_-chimeras co-expressed with β_1_-K732E. For B-D, Fn or 9EG7 binding was measured with HEK293FT-α_5_β_1_-KO cells transfected with the indicated α_5_β_1_ constructs plus GFP or GFP-TH. β_1_-K732E was used to increase the sensitivity of the assay. The GFP and integrin double-positive cells were analyzed. Data are presented as the MFI of the ligand or mAb normalized to integrin expression, and shown as mean ± s.e.m. (n ≥ 3). Two-tailed Student’s t-tests were performed between α_5_-chimeras and α_5_-WT, or as indicated under the same conditions. *P < 0.05; **P < 0.01; ***P < 0.001; n.s., not significant.

Twelve integrin α subunits share the integrin β_1_ subunit, making the largest β_1_ integrin subfamily (Fig. 1A). The major fibronectin receptor, integrin α_5_β_1_, has been relatively well studied structurally and functionally. Like α_IIb_β_3_ and α_L_β_2_, the α_5_β_1_ integrin can be activated by the overexpression of TH^20^. We have demonstrated the important role of the CTMD regions of α_IIb_, α_V_, and α_L_ subunits in TH-induced integrin activation^13^. Here, we extended our study to α_5_β_1_ integrin and asked whether the α_5_ CTMD region follows the same rule. Using the α_5_β_1_-deficient CHO-B2 cell line^21^, we found that complete deletion of the α_5_ CTMD region abolished GFP-TH-induced binding of fibronectin fragment Fn9–10 to α_5_β_1_ integrin (Fig. 8A), demonstrating the requirement of α_5_ CTMD region in α_5_β_1_ inside-out activation. The next question is whether the diverse α CTMD regions of the β_1_ integrin family also differently regulate β_1_ integrin activation. To answer this question, we compared the function of all the α CTMD regions of the β_1_ integrin family in the context of α_5_ subunit. Eleven α_5_ chimeras were generated by replacing the α_5_ CTMD region with that of α subunits as indicated in Figs 2A and 8B. The TH-induced fibronectin-binding assay for α_5_β_1_ was performed using the α_5_β_1_-knockout HEK293FT cells. The activating β_1_-K732E mutation, located at the transmembrane domain^22^, was used to enhance the sensitivity of the assay. As shown in Fig. 8B, the β_1_-K732E significantly increased TH-induced fibronectin binding compared with the β_1_ WT. Among the α_5_ chimeras, two groups were identified: one group has no but the other has significant effect on TH-induced fibronectin binding compared with the α_5_ WT (Fig. 8B). Remarkably, all the α_5_ chimeras, including α_5_-α_4_, α_5_-α_9_, α_5_-α_3_, and α_5_-α_6_, which have a tyrosine residue adjacent to the GFFKR motif (Fig. 1A), showed comparable fibronectin binding with the α_5_ WT that also contains a tyrosine at the CTMD region (Figs 1A and 8B). In contrast, all the α_5_ chimeras, including α_5_-α_1_, α_5_-α_7_, α_5_-α_8_, and α_5_-α_10_, α_5_-α_11_, and α_5_-α_V_, which lack the tyrosine residue adjacent to the GFFKR motif (Fig. 1A), rendered α_5_β_1_ more active than the WT (Fig. 8B). However, an exception is the α_5_-α_2_ chimera, which has an equivalent tyrosine at the α_2_ CTMD region but significantly increased α_5_β_1_ activation (Figs 1A and 8B). The similar results were obtained when the selected α_5_ chimeras were co-expressed with the β_1_ WT in the TH-induced fibronectin binding assay (Fig. 8C). The α_5_-α_2_ and α_5_-α_10_ significantly increased α_5_β_1_ activation while the α_5_-α_4_ had no obvious effect (Fig. 8C). By contrast, the enhanced effect of α_5_-α_7_ was not detectable when the β_1_ WT was used (Fig. 8C), indicating the low sensitivity of the assay. Finally, we correlated the TH-induced α_5_β_1_ activation with the large-scale conformational change of β_1_ ectodomain using the active conformation dependent mAb 9EG7. The presence of β_1_-K732E significantly enhanced the TH-induced 9EG7 binding. Consistent with the fibronectin binding assay, the α_5_-α_2_, α_5_-α_7_, and α_5_-α_10_ significantly increased 9EG7 binding, but the α_5_-α_9_ had no such effect compared with the α_5_ WT (Fig. 8D). These data suggest that the α CTMD regions of the β_1_ family could contribute differently to β_1_ integrin inside-out activation. They may also follow the rule of the negative regulation by a tyrosine residue.

## Discussion

Compared with the extensive structural and functional studies of the relatively conserved β integrin CT that serve as docking sites for many signaling molecules, little is known about the role of α integrin CT especially the non-conserved MD regions. Since many α integrin subunits share the same β subunit, it is tempting to speculate that on one side the α CTMD regions may be interchangeable; on the other side, they may provide the specificity for integrin function. One of the difficulties in studying the α integrin CTMD regions is the sequence diversity among 18 α subunits. Another challenge is the lack of well-established activation assays for many integrin members, which limits the functional studies of the CTMD regions for many α integrins. Our approach in the current study provides a useful tool to thoroughly examine the potential functions of the CTMD regions of almost all integrin α subunits. By putting the different α CTMD regions in the context of the α_IIb_, α_L_, or α_5_ subunit, this approach made it possible to compare the function of different α CTMD regions.

The potential function of several individual α integrin CTMD regions in integrin activation had been indicated in previous studies more than 20 years ago. They showed that deletion of the α CTMD region diminished cell adhesion or migration mediated by α_1_, α_2_, α_4_, α_V_, and α_6_ integrins^23–30^ and dampened PMA-induced activation of α_L_β_2_ integrin^31^. Direct evidence for a role of α CTMD region in integrin inside-out activation was provided by the observation that complete deletion of the CTMD region of α_IIb_, α_V_, or α_L_ integrin abolished talin and kindlin-mediated integrin ligand binding and conformational changes^13,14^. It was suggested that the presence but not the sequence of specific residues was required for the α CTMD region to support talin-induced integrin activation^14^. In the current study, we found that replacing the α CTMD region of α_IIb_, α_L_, or α_5_ integrin with those of other α integrins still maintained the capability of integrin inside-out activation mediated by the overexpression of TH, suggesting that the α CTMD region can be interchangeable for this common activation supportive function. However, we observed significant variations of the activation levels among the same α integrins carrying different CTMD regions, indicating that the α CTMD region also plays a regulatory role in integrin inside-out activation.

We found that replacing the α_IIb_ CTMD region with that of α_V_ integrin markedly reduced TH-induced activation of α_IIb_β_3_. By contrast, the α_1_ and α_L_ CTMD regions rendered α_IIb_β_3_ more active than WT, consistent with the previous observation that replacing the α_IIb_ CT with those of α_2_, α_5_, α_6A_, or α_6B_ that do not natively pair with β_3_ subunit enhanced α_IIb_β_3_ activation despite they share the same GFFKR motif ^32^. Thus, the native pair between the α CTMD region and β CT is important to maintain the resting state of integrin. Although the α_V_ CTMD region shares six consensus residues with α_IIb_ CTMD region, which is half of the length of α_IIb_ CTMD region (Fig. 1A), it exerts different effect on α_IIb_β_3_ activation. A predicted β turn structure formed by the PPQEE motif of α_V_ CTMD region was suggested to regulate the conformation and ligand binding of α_V_β_3_^26^. A similar motif PPLEE was also found in the α_IIb_ CTMD region (Fig. 1A). Peptides containing this motif of α_IIb_ or α_V_ CTMD region could inhibit the activation of α_IIb_β_3_ or α_V_β_3_^33^, indicating that it could not be the reason for the different regulation by the α_IIb_ and α_V_ CTMD regions. Although the structure of the α_IIb_ CT has been determined, it shows large conformational variations (Fig. 1B). It is not known if the structural flexibility of α_IIb_ CT is functionally relevant. The α_IIb_ CTMD region has a unique tandem acidic residue motif, EEDDEEGE, which is conserved among the α_IIb_ from different species and not seen in the α_V_ CTMD region (Fig. 1A and data not shown). These negatively charged residues might regulate the conformation of α_IIb_ CT through repulsive interactions with the acidic phospholipid head groups at the cytosolic face of cell membrane, or through ionic interactions with the positively charged residues at the membrane-proximal region of α_IIb_ CT as suggested by a NMR study^34^. The membrane-permeable peptides containing the α_IIb_ CTMD region were shown to block α_IIb_β_3_ activation in platelets^15,35^, and to inhibit the association of talin with α_IIb_β_3_ in thrombin-activated platelets^36^. In addition, the TH domain contains several positively charged surface residues that have been shown to be important for its integrin activating function through interacting with the cell membrane^37–39^. It is possible that the negatively charged α_IIb_ CTMD region may also affect the orientation of TH domain when TH encounters with β_3_ CT, but a direct interaction between TH and α_IIb_ remains to be confirmed. Being critical in hemostasis and thrombosis, the activation of α_IIb_β_3_ is strictly regulated in platelets^40^. Our data demonstrated that the unique feature of α_IIb_ CTMD region renders α_IIb_β_3_ more susceptible to the signals of inside-out activation, which is in line with the high extent of activation required for α_IIb_β_3_ function.

The lymphocyte-specific α_L_β_2_ is another integrin whose activation is highly regulated by the inside-out signals^5,41^. The α_L_ CT has the second longest MD region that folds into α-helical conformation as shown in an NMR structure (Fig. 1A,B). Our recent study showed that deletion of the α_L_ CTMD region completely abolished TH-induced ICAM-1 binding to α_L_β_2_ integrin^13^. Here we found that replacing the α_L_ CTMD region with that of α_X,_ α_D_, or α_M_ all significantly reduced ICAM-1 binding mediated by TH. This was not determined by the differences in the length but by a common tyrosine residue seen in the α_X,_ α_D_, and α_M_ CTMD regions. Remarkably, we found that the position of the tyrosine at the α CTMD region is important to exert the negative effect on integrin inside-out activation. The bulky side chain and the hydroxyl group of tyrosine are critical for this regulatory function. Interestingly, the same negative effect was observed when introducing the tyrosine mutations into the equivalent position of the α_IIb_ CTMD region^13^. One caveat of our study is that the negative effect of the tyrosine was found in the context of α_L_ integrin in which no native tyrosine is present in the CTMD region. However, the physiological relevance of our discovery was built on the same observations on the α_M_β_2_ integrin, in which mutating the native tyrosine residue greatly enhanced the inside-out activation of α_M_β_2_, suggesting that the tyrosine residue negatively regulates the activity of α_M_β_2_. The tyrosine residue is also conserved among the α_M_, α_X_, or α_D_ integrins from different species (data not shown). Indeed, in contrast to the α_L_β_2_ integrin that only binds selectively to ICAMs, the α_M_β_2_, α_X_β_2_, and α_D_β_2_ integrins all exhibit multiligand-binding properties^42–44^. The negative regulation by a tyrosine residue in their α CTMD regions may exert a restraint to avoid hyperactivity (or to balance the activation) of these β_2_ integrins, in accordance with their less-selective ligand binding functions.

Several studies have provided evidence demonstrating that the activation of α_L_β_2_ and α_M_β_2_ are differently regulated. Different chemokines and chemoattractants were shown to stimulate inside-out activation of α_L_β_2_ and α_M_β_2_, which was suggested to be mediated by distinct pathways via the α CTs^45,46^. Given the β_2_ subunit shares the common integrin activation pathway mediated by talin and kindlin, other signaling molecules may be involved in the different regulation of α_L_β_2_ and α_M_β_2_ probably through direct interaction with the α CT. An example is the Rap-1 interacting effector molecule RapL that specifically binds to the α_L_ CTMD region to support Rap-1-mediated α_L_β_2_ activation^47^. It was known that the α_L_-K1097 is involved in RapL binding^48^. Our results show that the α_L_-K1097Y mutation rendered α_L_β_2_ the least active among the tyrosine mutations tested. However, RapL is predominantly expressed in immune cells^49^. It should not account for the decreased activation by the α_L_-K1097Y mutation since integrin activation was measured in HEK293FT cells lacking RapL expression. A Ser phosphorylation was found in the CTMD regions of both α_L_ and α_M_ subunits^50,51^. However, mutations of the Ser residue only blocked the conformational changes involved in α_M_β_2_ but not α_L_β_2_ activation, indicating different regulation by the α_M_ CT. Here, we identified a tyrosine residue at the α_M_ CTMD region that is also involved in the specific regulation of α_M_β_2_ inside-out activation.

It remains unknown whether the activation of β_1_ integrin family members are all subjected to inside-out regulation. Recent structural studies demonstrated that the conformational activation of α_5_β_1_ integrin could be modulated by many components including the transmembrane and cytoplasmic domains^52–55^. We found that similar to α_IIb_, α_V_, and α_L_ integrins the α_5_ CTMD region is also required for TH-induced inside-out activation, rationalizing the use of α_5_β_1_ as a model integrin to study the function of α CTMD regions. Our studies on all the α CTMD regions of the β_1_ subfamily suggest a potential regulatory function of these regions in β_1_ integrin inside-out activation. Remarkably, a tyrosine residue at the α CTMD region seems to play a similar negative role as seen in α_M_ integrin in regulating α_5_β_1_ inside-out activation. All the α CTMD sequences lacking a tyrosine proximal to the GFFKR motif promoted α_5_β_1_ inside-out activation, while the α CTMD sequences having the tyrosine had no such effect, with the exception of α_2_ CTMD region. Notably, the α_7_ CTMD region contains a tyrosine at the 11^th^ position distal from the GFFKR motif (Fig. 1A), but the α_5_-α_7_ exhibits a high level of integrin activation, consistent with the hypothesis that the membrane-proximal location of the tyrosine is important to exert an inhibitory effect. Interestingly, the α_10_ CTMD region that is abundant in negatively charged residues as seen in α_IIb_ CTMD region exerted the most dramatic effect on α_5_β_1_ activation. Consistently, the α_V_ CTMD region increased α_5_β_1_ activation due to the lack of the tyrosine residue when compared with α_5_ WT, but it decreased α_IIb_β_3_ activation due to the lack of the cluster of acidic residues when compared with α_IIb_ WT. We found that α_2_ CTMD region rendered α_5_β_1_ more active despite having the consensus tyrosine, suggesting that other residues of the α_2_ CTMD region may counteract with the negative effect of tyrosine in regulating integrin activation.

Our study raised the question of the mechanism by which the α CTMD regions contribute to integrin inside-out activation. Previous and our current data demonstrated that the integrin α CTMD region is involved in the associations at the α/β transmembrane and CT domains, required to maintain integrin in the resting state^13^. In line with this function, our current data suggest two functional aspects of α CTMD region in integrin inside-out activation, i.e. the activation-supportive function and the activation-regulatory function. Structure analysis suggested that when binding to the β_3_ CT, the talin 1 head domain might encounter steric hindrance with the α_IIb_ CTMD residues immediately following the GFFKR motif^13,14,56–58^. Such interactions may disrupt the integrin α/β association at the cytoplasmic as well as transmembrane domains, leading to an active ectodomain conformation capable of high-affinity ligand binding. This non-specific interaction is required for the activation-supportive function of α CTMD region, which could be independent of the amino acid sequences. In addition, a minimal length of two amino acids of the α CTMD region could support the integrin inside-out activation although at a reduced level^13^. This model is consistent with our observation that the α CTMD regions are interchangeable for the activation-supportive function that is not sensitive to the diversities of sequence and length.

In contrast, the activation-regulatory function of α CTMD region is dependent on certain amino acids. Several potential mechanisms are involved in this regulation. We found that the differences of the α CTMD regions in regulating integrin activation were not due to the effect of talin binding to the β CT since truncation or swapping the α CTMD region did not reduce the amount of TH bound to the β CT^13^. The specific amino acids of the α CTMD region may directly affect the conformational change of α cytoplasmic as well as transmembrane domains induced by the binding of integrin activators such as talin and kindlin. Recent studies provided evidence suggesting the conformational change of α_IIb_ transmembrane and cytoplasmic domains in the context of full-length integrin on the cell surface^59^. In addition, different levels of integrin activation had been observed when introducing mutations into the transmembrane or cytoplasmic domains, which correlate with the different levels of ectodomain conformational changes (unpublished data). Certain amino acids such as the acidic residue clusters present in α_IIb_ and α_10_ CTMD regions and the tyrosine present in a subset of α CTMD regions may either facilitate or restrain the conformational change of α subunit as demonstrated by the active conformation-specific mAbs. The regulation of receptor activity by a tyrosine residue at the cytoplasmic domains has been seen in many cell surface receptors^60^. It was proposed that the tyrosine residue could be buried in the cell membrane to restrain the movement of the cytoplasmic domain in the resting state. Such restraints could be released upon the tyrosine phosphorylation. This mechanism may also be applied to the specific tyrosine of α integrin CTMD regions. Structures of α_4_, α_M_, and α_X_ CT indicate that the tyrosine could be buried in the cell membrane (Fig. 1B). The tyrosine may restrain the piston like movement of α CT or the conformational plasticity of the GFFKR region as suggested by structural studies^59,61–63^. This negative effect depends on the position of tyrosine as shown by our current data. Whether the tyrosine can be phosphorylated to release its negative effect on integrin activation clearly requires further investigation on individual integrins. Another potential mechanism by which the α integrin CTMD regions regulate the levels of integrin activation is through their interacting proteins. Several α integrin CT binding proteins have been identified to function as either negative or positive regulators for integrin activation^8,64^. Some of the regulators such as SHARPIN, MDGI, and filamin interact with a subset of α integrins while the others bind to specific α integrins, such as Nischarin for α_5_ and CIB1 for α_IIb_^54,62,65–67^. Interestingly, most of the current α CT binding proteins interact with the membrane-proximal region containing the conserved GFFKR motif ^8^. More novel integrin activation regulators interacting with the α CTMD region are yet to be identified.

There are accumulating data showing that the diverse α integrin CTMD regions can specify the cellular function of integrins. Novel functions of the α CTMD regions have been identified in recent years. For example, a subset of α integrin CTMD regions was found to regulate integrin internalization through interacting with the endocytic clathrin adaptor AP2^18^. Specific interaction between integrin α_5_ CTMD region with phosphodiesterase-4D5 (PDE4D5) was found to regulate endothelial inflammatory signaling^68^. It is tempting to speculate that more novel functions of α integrin CTMD regions are yet to be identified. Our large-scale analysis of the function of α integrin CTMD regions provokes new hypotheses that need to be tested on individual integrins. In addition, our approach provides a valuable tool and resource to study the integrin signaling events that are specified by the α CTMD regions.

## Materials and Methods

### DNA constructs

Plasmid DNA constructs for human α_IIb_β_3_, α_L_β_2_, α_5_β_1_, and EGFP-tagged mouse talin-1-head (GFP-TH) were as described^20,69,70^. Mutations were introduced by PCR following the protocol of the QuikChange XL site-directed mutagenesis kit (Agilent Technologies). α_5_-CRISPR/Cas9 and β_1_-CRISPR/Cas9 plasmids were purchased from Santa Cruz Biotechnology. Human ICAM-1 cDNA was obtained from Addgene. The cDNA of ICAM-1 extracellular domain was amplified by PCR and subcloned into a modified pIRES2-EGFP vector with a tag of human IgG1 Fc region at the C-terminus (denoted as ICAM-1-Fc).

The integrin α_IIb_-chimeras were generated by the overlap PCR to replace the cDNA of α_IIb_ CTMD region with the cDNA of the CTMD region of α_V_, α_1_, or α_L_ integrin. The chimeric full-length α_IIb_ cDNA was cloned into the pEF1/V5-HisA vector using the 5′ EcoRV and 3′ XbaI restriction sites. A stop codon was added right before the 3′ XbaI site. The integrin α_L_-chimeras were constructed using the 5′ Bsp1407I site right before the cDNA of KVGFFKR motif and the 3′ XbaI site preceded by a stop codon. The extra Bsp1407I and XbaI sites in the WT α_L_ vector were silenced by site-directed mutagenesis. A set of sense and antisense overlapping primers were designed to encode the sequence of KVGFFKR followed by the CTMD region of α_D_, α_M_, or α_X_ integrin. The 5′ Bsp1407I and 3′ XbaI sites as well as a stop codon were included in the primers. The chimeric cDNA fragments were obtained by mixing the primers for PCR amplification and subcloned into the α_L_ construct using the Bsp1407I and XbaI sites. The full-length cDNA of human α_M_ integrin was cloned into the pcDNA3.1 vector using the 5′ KpnI and 3′ XbaI sites. Mutations at the α_M_ CTMD region were introduced by PCR. The integrin α_5_-chimeras was constructed using a 5′ HindIII site preceding the GFFKR-coding sequence and a 3′ MluI site preceded by a stop codon. A set of sense and antisense overlapping primers were designed to encode the sequence of GFFKR followed by the CTMD region of α_V_, α_1_, α_2_, α_3_, α_4_, α_6_, α_7_, α_8_, α_9_, α_10_, or α_11_ integrin. The chimeric cDNA fragments were generated by PCR and subcloned into the WT α_5_ vector. All the DNA constructs were validated by DNA sequencing.

### Antibodies, inhibitors and ligands

PAC-1 (BD Biosciences) is a ligand-mimetic IgM mAb that is specific to activated α_IIb_β_3_^71^. AP3 is non-blocking anti-β_3_ mAb^72^. mAb 370.3 is specific for the extended conformation of α_IIb_^13,70^. PE-labeled or unlabeled TS2/4 (BioLegend) is non-blocking anti-α_L_ mAb. m24 (Biolegend) and KIM127 are anti-β_2_ conformation-specific mAbs that report β_2_ integrin headpiece opening and extension, respectively^19,73–75^. 2LPM19c is an anti-α_M_ mAb (Santa Cruz Biotechnology). PE-labeled MAR-4 (BD Biosciences) is a non-blocking anti-β_1_ mAb. 9EG7 (BD Biosciences) is a rat anti-β_1_ conformation-specific mAb that reports β_1_ integrin extension^76^. Rabbit anti-GFP antibody was from Immunology Consultants Laboratory. Rabbit anti-β_3_ antibody (H-96) was from Santa Cruz Biotechnolgy. Anti-β-actin mAb was purchased from Sigma. 9EG7 were conjugated with Alexa Fluor-647 (Life Technologies). AP3 was conjugated with R-PE using the R-PE antibody conjugation kit (Solulink). 370.3, m24, and KIM127 were biotinylated using the EZ-Link Sulfo-NHS-Biotin (Thermo Scientific). A286982 (Santa Cruz Biotechnology) is a α_L_β_2_-specific inhibitor. Eptifibatide acetate (Santa Cruz Biotechnology) is a α_IIb_β_3_-specific inhibitor.

Human ICAM-1-Fc was expressed as the secreted form in HEK293FT cells via transient transfection using polyethylenimine (PEI). The transfected cells were cultured for 10 days before the culture supernatant was collected. The concentration of ICAM-1-Fc in the supernatant was determined by ELISA using the anti-human ICAM-1 mAb (SinoBiological, Inc.) and the peroxidase-conjugated anti-human IgG1 (Fc specific) (Jackson ImmunoResearch Laboratories, Inc.). The culture supernatant was used for the ICAM-1 binding assay. Human fibronectin type III domains 9^th^−10^th^ fragment (Fn9–10) was expressed in *E*. *coli* and purified as described before^77^. Human fibronectin (Fn) was purchased from Sigma. Fn9–10 and Fn were conjugated with Alexa Fluor-647.

### Cell lines

HEK293FT cells (ThermoFisher Scientific) were cultured in DMEM plus 10% FBS at 37 °C supplied with 5% CO_2_. The α_5_ and β_1_ integrin double-knockout HEK293FT (HEK293FT-α_5_β_1_-KO) cells were generated by the CRISPR/Cas9 gene editing technology as described in our previous study^78^. α_5_β_1_ integrin deficient CHO-B2 cells were as described before^21^.

### Soluble ligand binding assay by flow cytometry

GFP-TH induced ligand binding assay of HEK293FT cells transfected with α_IIb_β_3 or_ α_L_β_2_ integrin was as described previously^13^. In brief, HEK293FT cells were co-transfected with integrin constructs and GFP or GFP-TH for at least 24 hours. Ligand binding was performed in HBSGB buffer (25 mM HEPES pH 7.4, 150 mM NaCl, 2.75 mM glucose, and 0.5% BSA) plus 10 μM eptifibatide acetate (for α_IIb_β_3_), 50 μM A286982 (for α_L_β_2_), or 1 mM Ca^2+^ and 1 mM Mg^2+^ (Ca/Mg). The cells were first incubated at 25 _°_C for 30 min with 5 μg/ml PAC-1 and 10 μg/ml biotinylated AP3 for α_IIb_β_3_, 42 μg/ml of ICAM-1-Fc and 45 μg/ml biotin-conjugated goat anti-human IgG Fc (Novex, Life Technologies) for α_L_β_2,_ and then washed and incubated in Ca/Mg on ice with the detecting reagents: PE-labeled streptavidin and Alexa Fluor-647-labeled goat anti-mouse IgM for α_IIb_β_3_, PE-labeled TS2/4 and Alexa Fluor 647-labeled streptavidin for α_L_β_2_. For α_5_β_1_ integrin, either CHO-B2 or HEK293FT-α_5_β_1_-KO cells were transfected with α_5_β_1_ integrin plus GFP or GFP-TH. The cells were first incubated with 50 μg/ml Alexa-Fluor-647-labeled Fn9–10 or 15 μg/ml Alexa-Fluor-647-labeled Fn in HBSGB buffer plus 5 mM EDTA or 1 mM Ca/Mg, and then washed and incubated in Ca/Mg on ice with PE-labeled MAR-4. Integrin and GFP double-positive cells were acquired for calculating the mean fluorescence intensity (MFI) by flow cytometry. Ligand binding was presented as the normalized MFI, that is ligand MFI (after subtracting the ligand MFI in the inhibitor or EDTA condition) as a percentage of integrin MFI.

### Conformation-specific antibody binding

Integrin conformational changes detected by the conformation-specific mAbs on the cell surface was as described previously^13,70^. In brief, HEK293FT transfectants were first incubated with the biotinylated conformation-specific mAb in Ca/Mg at 25 _°_C for 30 mins, washed and then incubated with the detecting reagents: Alexa-Fluor-647-labeled streptavidin plus R-PE-labeled AP3 for α_IIb_β_3_ or PE-labeled TS2/4 for α_L_β_2_. For α_5_β_1_ integrins, HEK293FT-α_5_β_1_-KO transfectants were incubated with rat 9EG7 mAb in Ca/Mg at 25 _°_C for 30 mins, washed and then incubated with PE-labeled MAR-4 and Alexa-Fluor-647-labeled goat anti-rat IgG (cross-absorbed) (Abcam). Integrin and GFP double-positive cells were analyzed for calculating the MFI of mAb binding. The binding of conformation-specific mAb is presented as their MFI normalized to the MFI of integrin expression.

For m24 binding with α_M_β_2_ integrin, HEK293FT transfectants were incubated with m24 or mouse anti-α_M_ mAb (2LPM19c) separately, washed and incubated with Alexa-Fluor-647-labeled anti-mouse IgG. GFP-positive cells were analyzed for calculating the MFI of bound m24 or 2LPM19c. The m24 binding was presented as the m24 MFI normalized to the 2LPM19c MFI reporting α_M_β_2_ expression.

### Co-immunoprecipitation

To detect the TH-binding to integrin β_3_ CT, the α_IIb_β_3_ constructs were co-transfected with GFP-TH or GFP into HEK293FT cells. The cells were lysed to perform co-immunoprecipitation with rabbit anti-GFP antibody. The GFP-TH associated β_3_ subunit was detected by immunoblot using rabbit anti-β_3_ antibody H-96 and HRP-labeled anti-rabbit IgG antibody. The amount of GFP or GFP-TH in pull-down samples was detected by rabbit anti-GFP antibody and HRP-labeled anti-rabbit IgG antibody. Total expression levels of integrin β_3_ and GFP-TH in the whole cell lysates were detected by immunoblot. β-actin was blotted with anti-β-actin mAb as a loading control.

To detect the TH binding to integrin β_2_ CT, the α_L_ constructs were co-transfected with β_2_-D709A and GFP-TH into HEK293FT cells. GFP-TH only or α_L_β_2_ plus GFP transfectants were used as negative controls. The cells were washed with PBS at pH 8.0 and the cell surface molecules were biotinylated using 2 mM biotin reagent (EZ-Link™ Sulfo-NHS-Biotin, Thermo Scientific) in PBS at 25 °C for 30 min. The cells were washed and lysed for co- immunoprecipitation with anti-GFP antibody. As a positive control for integrin α_L_ and β_2_ bands, α_L_β_2_ was immunoprecipitated with anti-α_L_ mAb TS2/4. Integrin α_L_ and β_2_ subunits that associated with GFP-TH were quantitatively detected by blotting with IRDye® 800CW Streptavidin (LI-COR). The immunoprecipitated GFP or GFP-TH were detected by Western blot using anti-GFP antibody. Integrin α_L_ and β_2_ signals were normalized to the precipitated GFP or GFP-TH, and shown as a percentage of the normalized α_L_-WT signal. The cell surface expression of α_L_β_2_ integrins and GFP-TH were detected by flow cytometry.

### Statistical Analysis

Data are expressed as mean ± s.e.m from at least three independent experiments (n ≥ 3 in each group) unless specified. Statistical analyses were performed with GraphPad Prism using parametric Student’s t-test (two-tailed).

### Data Availability

The datasets generated during and/or analyzed during the current study are available from the corresponding author on reasonable request.

## Electronic supplementary material

Supplementary figure
