# Supplementary material for: The membrane-distal regions of integrin α cytoplasmic domains contribute differently to integrin inside-out activation
Source: Sci Rep. 2018 Mar 22;8:5067. doi: 10.1038/s41598-018-23444-w (PMC5864728; doi:10.1038/s41598-018-23444-w)
Supplement: Supplementary file 1 — Supplementary figure [file 41598_2018_23444_MOESM1_ESM.docx]

The-membrane distal regions of integrin α cytoplasmic domains contribute differently to integrin inside-out activation

**Aye Myat Myat Thinn^1,2^, Zhengli Wang^1^, and Jieqing Zhu^1,2,*^**

^1^Blood Research Institute, BloodCenter of Wisconsin, Milwaukee, WI 53226

^2^Department of Biochemistry, Medical College of Wisconsin, Milwaukee, WI 53226

*To whom correspondence should be addressed: Jieqing Zhu, Ph.D. Blood Research Institute, BloodCenter of Wisconsin, 8727 Watertown Plank Road, Milwaukee, WI 53226. Telephone: 414-937-3867; FAX: 414-937-6284; E-mail: [Jieqing.Zhu@bcw.edu](mailto:Jieqing.Zhu@bcw.edu)

**Supplementary Figure**


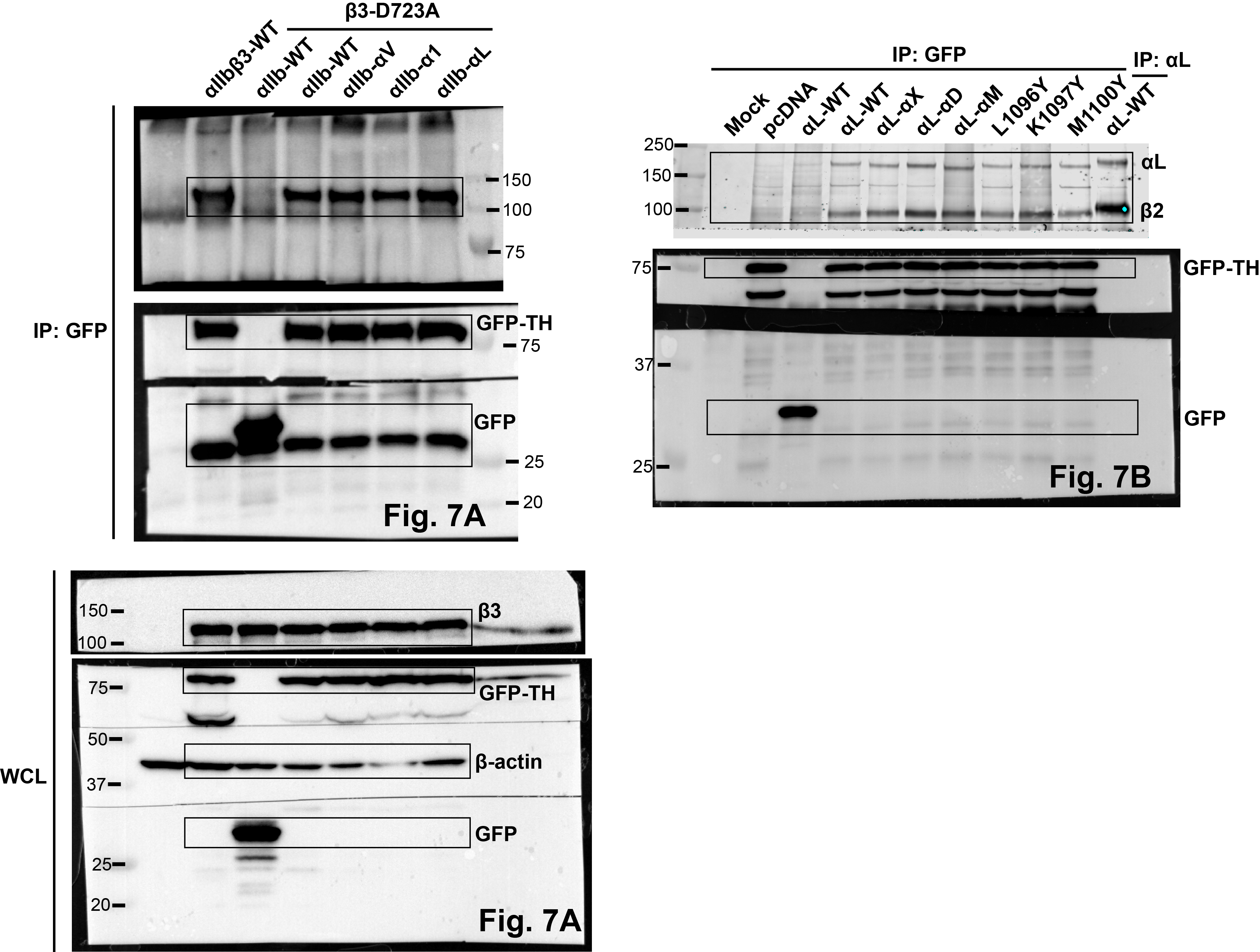


**Figure S1**. Full-length western blots for figures 7A and 7B. The IP samples in Fig. 7A were run on the same gel and transferred to the PVDF membrane. The membrane was blotted with rabbit anti-β3 (H96). The membrane around 50kDa was cut out to eliminate the strong signals from the heavy chain of rabbit anti-GFP antibody that was used for IP. The same membrane was cut around 75kDa and 37-17kDa for GFP-TH and GFP bands, respectively, before re-blotting with rabbit anti-GFP antibody. The WCL samples in Fig. 7A were run on a separate gel and transferred to the PVDF membrane. The membrane was cut and blotted separately for indicated proteins. All the membranes were scanned with the Image Quant LAS 4000 using automatic exposure. The IP samples in Fig. 7B were run on the same gel and transferred to the PVDF membrane. The membrane was cut and blotted separately. The upper membrane was blotted with streptavidin-780CW and scanned with the Odyssey imager. The lower membrane was blotted with rabbit anti-GFP antibody for GFP-TH and GFP bands. The membrane around 50kDa was cut out before scanning with the Image Quant LAS 4000 to eliminate the strong signals from the heavy chain of rabbit anti-GFP antibody that was used for IP.
